# Giant Duckweed (*Spirodela polyrhiza*) Root Growth as a Simple and Sensitive Indicator of Copper and Chromium Contamination

**DOI:** 10.3390/toxics11090788

**Published:** 2023-09-18

**Authors:** Hojun Lee, Jonas De Saeger, Sunwoo Bae, Mirae Kim, Stephen Depuydt, Philippe M. Heynderickx, Di Wu, Taejun Han, Jihae Park

**Affiliations:** 1 Bio Environmental Science and Technology (BEST) Lab, Ghent University Global Campus, 119-5, Songdomunhwa-ro, Incheon 21985, Republic of Korea; hojun.lee@ugent.be (H.L.); sunwoo.bae@ghent.ac.kr (S.B.); mirae.kim@ghent.ac.kr (M.K.); taejun.han@ghent.ac.kr (T.H.); 2 Department of Plant Biotechnology and Bioinformatics, Ghent University, Technologiepark 71, 9052 Ghent, Belgium; jonas.desaeger@ghent.ac.kr; 3 VIB Center for Plant Systems Biology, Technologiepark 71, 9052 Ghent, Belgium; 4 Erasmushogeschool Brussel, Quai de l’industrie 170, 1070 Anderlecht, Belgium; stephen.depuydt@ehb.be; 5 Center for Environmental and Energy Research, Ghent University Global Campus, 119-5, Songdomunhwa-ro, Incheon 21985, Republic of Korea; philippe.heynderickx@ghent.ac.kr (P.M.H.); di.wu@ghent.ac.kr (D.W.); 6 Department of Green Chemistry and Technology, Faculty of Bioscience Engineering, Ghent University, 653 Coupure Links, B-9000 Ghent, Belgium; 7 Department of Animal Sciences and Aquatic Ecology, Ghent University, Wetenschapspark 1, Bluebridge, 8400 Oostende, Belgium

**Keywords:** duckweed, growth, metals, root regrowth length, *Spirodela polyrhiza*

## Abstract

Aquatic environment are often contaminated with heavy metals from various industrial sources. However, physicochemical techniques for pollutant detection are limited, thus prompting the need for additional bioassays. We investigated the use of greater duckweed (*Spirodela polyrhiza*) as a bioindicator of metal pollution. We exposed *S. polyrhiza* to four pollutants (namely, silver, cadmium, copper, and chromium) and assessed metal toxicity by measuring its frond area and the length of its regrown roots. The plant displayed significant differences in both frond size and root growth in response to the four metals. Silver was the most toxic (EC_50_ = 23 µg L^−1^) while copper the least (EC_50_ = 365–607 µg L^−1^). Direct comparisons of metal sensitivity and the reliability of the two endpoint assays showed that root growth was more sensitive (lower in terms of 50% effective concentration) to chromium, cadmium, and copper, and was more reliable (lower in terms of coefficient of variation) than those for frond area. Compared to conventional *Lemna*-based tests, the *S. polyrhiza* test is easier to perform (requiring only one 24-well plate, 3 mL of medium and a 72-h exposure). Moreover, it does not require livestock cultivation/maintenance, making it more suitable for repeated measurements. Measurements of *S. polyrhiza* root length may be suitable for assessment when copper and chromium in municipal and industrial wastewater exceed the environmentally permissible levels.

## 1. Introduction

Bioassays are important tools for assessing water quality and for developing ecologically relevant safety standards for water management [1]. Chloroxygenic organisms (plants and protists) are frequently used as test species, highlighting the importance of primary producers for monitoring the functioning and health of ecosystems [2].

Since the 1930s, duckweeds (Lemnaceae) have been used extensively in fundamental and applied research in the environmental sciences, including in phytotoxicity testing and bioremediation [3,4]. Duckweeds have many useful characteristics as test organisms, including their small size, simple structure, high surface-to-volume ratio, rapid doubling time, genetic homogeneity, and relative ease of culturing (via asexual propagation) in the laboratory. Duckweeds are commonly found in fresh water and brackish ecosystems in temperate climates globally, where they serve as important food sources for various water birds and fish, as well as habitat for small invertebrates [5]. Of the five genera (*Landoltia* Les & D.J. Crawford, *Lemna* L., *Spirodela* Schleid, *Wolffia* Horkel ex Schleid, and *Wolffiella* Hegelm) and 57 species classified within the subclass Lemnoideae [6], most ecotoxicological studies have been performed using *Lemna*, particularly *L. gibba* (gibbous duckweed) and *L. minor* (lesser duckweed) [7]. *Lemna minor* is a model organism for OECD (Organization for Economic Co-operation and Development) [8] and ISO (International Organization for Standardization) test guidelines [9], partly because it is readily available in different parts of the world [10,11,12].

Greater duckweed (*Spirodela polyrhiza* (L.) Schleiden) has also been the subject of extensive physiological research [13]. In addition, *S. polyrhiza* had a high degree of genetic homogeneity based on DNA barcode analysis [14]. Compared to *Lemna* plants, the fronds of *S. polyrhiza* are typically larger (~8 mm in length compared to 1–6 mm for *Lemna*) [15,16], making them easier to handle and more suitable for repeated measurements.

*S. polyrhiza* plants respond to adverse conditions by forming a special starch-rich structure, turion, which sinks to the bottom of the water and remains inactive until the environment becomes favorable for the plant’s growth [17]. Turions germinate and develop new vegetative fronds from two meristematic pockets [18] when favorable growth conditions are encountered. Since turions can be stored for several months while maintaining a high germination rate, the use of turions as starting material for bioassays is highly advantageous. In addition, storing turions allows bioassay procedures to be delayed for an appropriate time [19]. Taking these practical characteristics into account, a *Spirodela* growth inhibition test using turion-derived fronds was recently standardized as ISO 20227: ‘Determination of the growth inhibition effects of waste waters, natural waters and chemicals on the duckweed *Spirodela polyrhiza*—Method using a stock culture independent microbiotest’ [20]. Baudo et al. [19] found comparable sensitivities between the two duckweed genera *Spirodela* and *Lemna* when exposed to herbicides (thifensulfuron-methyl, tribenuron-methyl, metribuzin, lenacil, tritosulfuron, linuron, terbutylazine, imazamox, and metamitron), inorganic and organic compounds (3,5-dichlorophenol, acetone, KCl, and ethanol), and metals (silver [Ag], copper [Cu], cadmium [Cd], nickel [Ni], mercury [Hg], cobalt [Co], chromium [Cr(VI)], and zinc [Zn]). In some cases, however, the reported effect levels of *Spirodela* tests for the same metal have been highly variable, highlighting the need to standardize the testing environment [21].

Standard *Lemna* bioassays are relatively straightforward. Plants are incubated in test vessels filled with growth medium at 25 °C under continuous illumination (usually 100 μmol m^−2^ s^−1^ photon flux density [PFD] light intensity) for 7 d (longer test periods increase potential interference from contamination), after which the inhibitory effects can be determined by measuring various endpoints, such as the number and size of fronds and wet or dry biomass [9].

A study carried out by Gopalapillai et al. [22] has demonstrated the ecological importance of root length as an appropriate endpoint for biomonitoring. The measurement of average root length was found to be more sensitive than other parameters, such as shoot size. It was the most reliable parameter with a low coefficient of variation compared to frond number or dry weight. In addition, the effect of chemicals was most predictable in the relationship between dose and root length. However, Park et al. [23,24] have developed a simpler protocol based on measurement of root regrowth in *Lemna* species. Compared to the conventional ISO 20079 procedure, our method is shorter in duration (3 d vs. 7 d for ISO 20079), requires only 3 mL (100–150 mL for ISO 20079) of test solution, and employs non-axenic plant material (axenic plant material for ISO 20079). In addition, inter-laboratory comparison tests based on the root regrowth of *L. minor* conducted by 10 international institutes showed 21.3% repeatability and 27.2% reproducibility for CuSO_4_ and 21.3% repeatability and 18.6% reproducibility for wastewater, complying with repeatability and reproducibility standards for bioassays as regulatory tools [24].

In the current study, we performed toxicity tests using *S. polyrhiza* and the conventional test species *L. minor* to evaluate their comparative sensitivities and reliabilities. We measured frond area and root elongation as endpoints and exposed plantlets to four metal pollutants (Ag, Cd, Cr, and Cu), considering the crucial role of these elements in the phytoremediation sector of polluted environments [25], where our study could provide a new contribution to research. We used the species sensitivity distribution to determine which of the two endpoints was appropriate for estimating the risk of metal toxicity to *S. polyrhiza*. We also compared the effective concentrations at which 50% inhibition occurs (EC_50_) for root length and frond area with the Korean Nationally Permissible Standard for Wastewater Discharges (NPSWD) [26].

## 2. Materials and Methods

### 2.1. Sample Collection and Maintenance

Mature *Spirodela polyrhiza* (L.) Schleiden plants were collected from the Upo wetland in Changnyeong-gun, Gyeongsangnam-do, Republic of Korea (35°32′40.128″ N, 128°25′8.04″ E). Fronds of *Lemna minor* THAN_L09 and collected *S. polyrhiza* were maintained in a 1.5-L glass tank with Steinberg medium [27] at 25 °C under continuous white light (30–40 µmol photons m^−2^ s^−1^) supplied by cool daylight fluorescent tubes (FL 20 SS/18D, Philips). The medium was replaced at 7-d intervals, and the pH was adjusted to 6.9 ± 0.2 by adding 1 M hydrochloric acid (HCl) or 1 M sodium hydroxide (NaOH) as needed.

### 2.2. Toxicity Testing

Toxicity tests were run in a controlled environment culture chamber at 25 ± 1 °C, pH 6.8–7.0, with continuous light (100 ± 10 µmol photons m^−2^ s^−1^), as described by Park [28]. The test solutions were not replaced during the exposure period (static test). We used a 24-well plastic plate (85.4 mm × 127.6 mm; 15.6 mm in diameter, SPL, Seoul, Republic of Korea) and 2.5 mL of test solution in each well. An individual rootless plantlet was added to each well with four plantlets per metal concentration and six concentrations per plate. Three replicate plates (n = 3) were exposed for 72 h. Different metal concentrations of the toxicants were generated by diluting the original stock solutions (1000 mg L^−1^) of AgNO_3_ (CAS No. 7761-88-8), CdSO_4_ (CAS No. 10124-36-4), K_2_Cr_2_O_7_ (CAS No. 7778-50-9), and CuSO_4_ (CAS No 7758-98-7) from Showa Chem. (Tokyo, Japan) in Steinberg medium. The stock solution of the test toxicant was stored in cool, dry conditions until the test solutions were prepared. The test dilutions were prepared in volumetric flasks and dispensed into the replicate test vessels, which were then left at room temperature for 1 h to allow the medium and toxicant to equilibrate. A fully randomized design was used to account for the variability in environmental conditions within the culture chamber. For our negative controls, we used identical culture mediums, test conditions, and procedures, but devoid of the test substance.

### 2.3. Measurement Methods

Healthy frond colonies of duckweed (dark green with two or three identical leaves attached) were selected for the experiment. Prior to exposure to the test solutions, roots were excised from fronds using stainless-steel scissors. Fronds were added to the wells under the conditions described by Park et al. [24,28]. After 72 h, the fronds were transferred to a glass slide with tweezers, and the upper part of each frond was attached to the glass. By wetting the fronds, the new roots could be easily straightened by careful manipulation with tweezers. The distance between the camera and the glass slide was adjusted and fixed. Images of the frond and regrown roots were analyzed using the ImageJ software (National Institutes of Health (NIH), Bethesda, MD, USA). Frond area (FA) and the length of the longest root (root length; RL) were measured for each plantlet.

### 2.4. Statistical Analysis

Analysis of variance (ANOVA) was performed using Microsoft Excel (2019) to test for statistical differences among treatments, with a significance level of *p* < 0.05. The data were first assessed to ensure that all statistical assumptions for ANOVA were met, including homogeneity of variances and normal distribution of the data. Subsequently, multiple comparison tests were carried out using the least significant difference (LSD) procedure. Average responses among treatments in each metal toxicity test were compared with one-way ANOVAs (*p* < 0.05). Results are reported as EC_50_, the effective concentration at which 50% inhibition occurs, with 95% confidence intervals (CIs) estimated by the linear interpolated method (ToxCalc 5.0, Tidepool Scientific, McKinleyville, CA, USA).

EC_50_ values from the frond and root tests were ordered by magnitude and coefficient of variation (CV) to approximate sensitivity (i.e., the lower the EC_50_ value, the more sensitive the endpoint) and the reliability (i.e., the lower the CV, the more reliable the endpoint), respectively. Mean rank values were calculated for each endpoint and metal.

Species sensitivity distribution (SSD) curves were fitted in R (R Core Team 2020) using the EC_50_ values derived from the dose-response curves based on the experiment or reported values for each metal species. From each SSD EC_50_ curve, the slope and two hazardous concentrations (HC_05_ and HC_50_) were derived numerically, which correspond to metal concentrations affecting 5% and 50% of the species in an assemblage, respectively.

The species sensitivity index (SSI) was then calculated as follows:

SSI = log_10_(HC_50_) − log_10_(EC_50_), (1)

SSI is a relative index of the difference in species sensitivity: A higher SSI indicates a higher sensitivity [29].

A predicted no-effect concentration (PNEC) was also estimated to assess the risk to the environment from exposure to hazardous chemicals released using the following equation [30]:

PNEC = HC_05_/AF, (2)

where HC_05_ is the concentration at which the no-observable-effect concentration (NOEC) is exceeded for 5% of the species sensitivity derived from the SSD, and AF is the assessment factor (10 is used to take into account the differences between laboratory conditions and natural conditions).

## 3. Results and Discussion

### 3.1. Silver Toxicity

**Figure 1:**
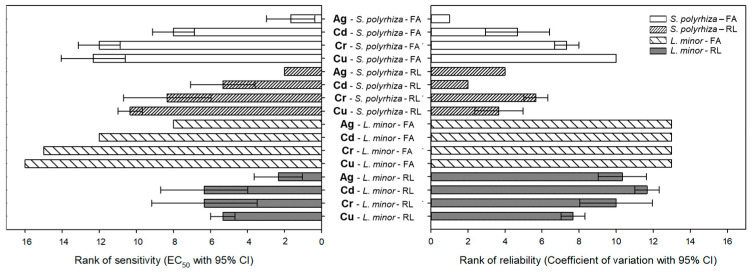
Mean ranks of sensitivity and reliability for each of the two endpoints (frond area and root regrowth length) in two duckweeds exposed to four metals (Ag, Cd, Cr, and Cu).

**Figure 2:**
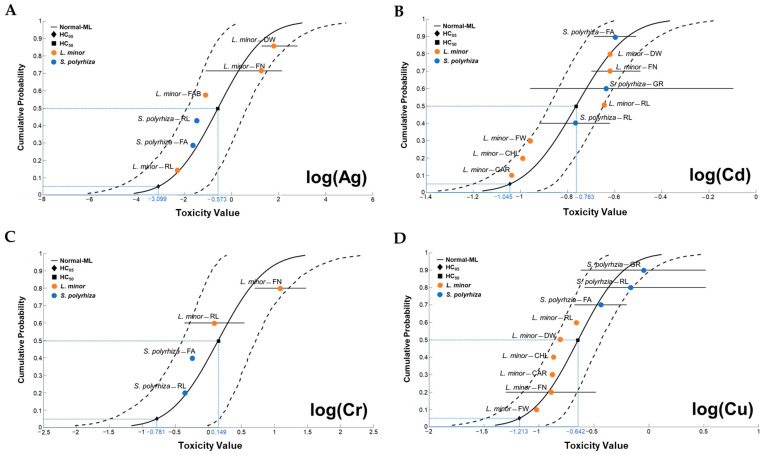
Species sensitivity distribution (SSD) curves for two duckweeds displaying (**A**) Log(HC_05_Ag_), (**B**) Log(HC_05_Cd_), (**C**) Log(HC_05_Cr_) and (**D**) Log(HC_05_Cu_). CAR, carotenoid content; CHL, chlorophyll content; DW, dry weight; FA, frond area; FAB, frond abscission; FN, frond number; FW, fresh weight; GR, growth rate; RL, root length. References [13,19,30,31,32,33,34,35,36,37,38].

Silver is known to cause frond abscission in *Lemna* and *Spirodela* and to inhibit frond and root proliferation [19,31,32,33,34]. In this study, the mean EC_50_ values for frond area (FA) and root length (RL) endpoints were 166 and 41 μg L^−1^ for *L. minor* and 23 and 23 μg L^−1^ for *S. polyrhiza*, respectively. In a previous study, EC_50_ values for *L. minor* ranged from 78 to 140 μg L^−1^, although these were based on growth rates inferred from frond number/biomass and photosynthetic pigment concentrations [31,34]. Park et al. [31] reported EC_50_ values of 5.3–37.6 μg L^−1^ for RL in *L. minor*. Baudo et al. [13] measured an EC_50_ value of 83 μg L^−1^ for FA in *S. polyrhiza*. Thus, the FA endpoint for *S. polyrhiza* in this study was 7.2 times more sensitive to Ag toxicity than *L. minor* and 3.6 times more sensitive than other measures for *Spirodela* from previous studies (Table 1). For *L. minor*, roots were more sensitive to Ag than fronds. By contrast, the response to Ag in *S. polyrhiza* was similar in both organs. The root sensitivity of *S. polyrhiza* to Ag was similar to that of *L. minor* (Figure 1 and Figure 2A).

### 3.2. Cadmium Toxicity

Cadmium has been shown to reduce cellular protein, carbohydrate, and chlorophyll contents in *Lemna*, as well as inhibit frond number, area, and biomass [32,34,35,36,37,38]. In the present study, EC_50_ of Cd toxicity for FA and RL measurements in *L. minor* were 415 and 155 μg L^−1^, respectively. The Cd EC_50_s for *S. polyrhiza* were 205 for FA and 122 μg L^−1^ for RL. Cd toxicity as measured by FA was two-fold higher in *S. polyrhiza* than in *L. minor* (Figure 1 and Figure 2B). Toxicity measured by RL did not differ between the species (Table 1). EC_50_ values measured by FA and RL in *S. polyrhiza* were comparable to those reported elsewhere in *Lemna* spp.

### 3.3. Chromium Toxicity

Lemnaceae plantlets appear to have a relatively high tolerance to Cr compared to other metals [39]. The EC_50_s for Cr toxicity measured by FA in *L. minor* and *S. polyrhiza* were 1756 and 507 μg L^−1^, respectively, and the EC_50_s for RL were 109 and 219 μg L^−1^ for each type of plantlet, respectively (Figure 2C). The Cr sensitivity of *S. polyrhiza* in the current study was higher than that of the same species reported by Baudo et al. (2130 μg L^−1^) [19]. The EC_50_ values for FA and RL inhibition in *S. polyrhiza* were similar to those of frond growth (584–35,000 μg L^−1^) and root growth (237.0–1148.3 μg L^−1^) in *Lemna* spp. (Table 1).

### 3.4. Copper Toxicity

Comparing the sensitivity of the two species to Cu for the relevant endpoints, the difference in FA between the two species is inconclusive based on the current data. For RL, *Spirodela* appears to be less sensitive than *Lemna*. However, previously reported EC_50_ values based on frond number and frond weight for *Lemna* spp. range from 160–616 μg L^−1^. On the other hand, Park et al. [31] calculated values between 221–470 μg L^−1^ for three *Lemna* species (*L. gibba*, *L. minor*, and *L. paucicostata*) when measuring RL. Based on these comparisons, the Cu sensitivity of *S. polyrhiza* is similar to that of *Lemna*, although it appears that roots are generally more sensitive to Cu than fronds (Figure 1 and Figure 2D).

### 3.5. Applications for Wastewater Management

For *S. polyrhiza,* the most sensitive assays (based on mean rank of the EC_50_ values) were measures of Ag toxicity using either RL or FA as endpoints. For *L. minor*, measuring Ag toxicity with RL was the most sensitive assay. The most reliable endpoint-metal combinations (based on the mean rank of the CV) were either FA-Ag or RL-Cd for *S. polyrhiza*. Other assays, including RL for Ag, Cd, Cr, and Cu, had intermediate levels of sensitivity for both species. Assays based on RL and FA (except FA-Ag) had moderate levels of reliability for *S. polyrhiza*.

Several of the toxicity assays for *Spirodela* tested here were able to detect pollutants at concentrations below the NPSWD in Republic of Korea. The EC_50_ values for Cr and Cu using RL were below NPSWD (500 μg L^−1^ and 3000 μg L^−1^, respectively) while only the EC_50_ for Cu was below NPSWD when using FA. The Korean Ministry of the Environment does not currently have a standard limit for Ag discharge. While both sensitivity and reliability are important criteria for evaluating laboratory tests, they must ultimately be compared to the costs and precision of in situ water quality tests.

In Republic of Korea, water quality monitoring is typically conducted via direct chemical analysis. This method has several drawbacks, including complex procedures for sample preparation, the need for expensive analytical equipment, and interference from secondary pollutants during analysis. To compensate for these shortcomings, the use of EC_50_ values obtained from bioassays should be considered to establish more ecologically meaningful permissible standards for wastewater discharge.

### 3.6. Predicted No-Effect Concentrations for Four Metals

The constructed SSD model used a log-normal distribution, and the simulation curves for the four metals in freshwater ecosystems are shown in Figure 1. In addition, to assess the potential risk to the aquatic environment, we compared the relevant literature for *Spirodela* and *Lemna* with the PNEC values derived from the SSD curves in this study with the metal tolerance limits in Korean river water as summarized in Table 2.

To ensure the safety of aquatic plants like duckweed from metal pollutants in their environment, the concentration of metals in the water (e.g., river water) where these organisms live must be kept below a specific threshold, i.e., the corresponding PNECs for these organisms. Currently, the allowable limits for metals in river water set by the Korean Ministry of Environment are registered as Cd < 5 μg L^−1^ and Cr(VI) < 50 μg L^−1^ (there are no criteria for Ag and Cu). Comparing the PNECs calculated from the results of the current study and the literature with the permissible levels of Cd and Cr shown in Table 2, it can be seen that the established limits for both Cd and Cr(VI) in river water are higher than the safety limits for the two aquatic plants. Therefore, the current management settings for Cd and Cr in river water need to be reviewed if *Spirodela* and *Lemna* are to survive as primary producers providing energy, food and nursery grounds for organisms of higher trophic levels.

## 4. Conclusions

Our newly developed phytotoxicity test using *Spirodela polyrhiza* has several advantages over conventional tests using *Lemna*. First, test material can easily be created by germinating turions from stock material, bypassing the need for considerable bench and incubation space associated with maintaining live *Lemna* stocks. In *S. polyrhiza*, the optimal conditions for germination and growth of dormant turions are well established. Second, the fronds and roots of *S. polyrhiza* are much larger than those of *Lemna* species, making them easier to handle and suitable for repeated measurements. Third, the sensitivity and reliability of toxicity assays using root growth in *S. polyrhiza* are comparable or superior to those of *Lemna*. The EC_50_ values measured by root growth inhibition were significantly lower than those of frond growth inhibition, providing further evidence that root length is a highly sensitive endpoint.

Finally, this new test can be performed in 24-well plates with 3 mL of growth medium per test sample and can be completed within 72 h. A good biological toxicity assay should be quick, easy to use, and sensitive to toxicants. In this respect, our 3-d test can be considered a modified version of the standardized 7-d test with *Lemna*. Test time is an important factor in selecting an appropriate bioassay as management decisions should be made promptly in the event of unpredictable pollution events. Both root length and frond area are sufficiently sensitive endpoints for detecting whether Cu or Cr levels exceed the permissible guidelines in Republic of Korea.

Overall, we showed the *Spirodela* root growth assay to be simple, rapid, inexpensive, and accurate for assessing the toxic risks of metals, especially relative to *Lemna*-based assays. We believe this technique has practical value for monitoring municipal and industrial wastewaters that commonly contain metal contaminants.

In the future, we will investigate the effects of these two metals on *S. polyrhiza* using different endpoints, such as: photosynthetic efficiency, pigment biosynthesis, reactive oxygen species (ROS) levels, and gene transcription, which will provide important insights into the relative sensitivity of different endpoints for assessing metal toxicity. In addition, they will shed light on the mechanisms of metal toxicity in a model aquatic macrophyte species.

## Figures and Tables

**Table 1:** Mean effective concentration values (EC_50_, EC_10_, NOEC, and LOEC; units: μg L^−1^) and the respective 95% confidence intervals (CI), obtained after 3 days of exposure to one of four metals (Ag, Cd, Cr, and Cu) in two duckweed species (*Spirodela polyrhiza* and *Lemna minor*).

Species Name	Parameters	Toxicity Values	Ag	Cd	Cr	Cu
*S. polyrhiza*	FA	EC_50_ (95% CI)	23.2 (18.8–25.9)	204.9 (113.3–292.5)	507.1 (261.5–818.4)	630.2 (227.0–1162.1)
		EC_10_ (95% CI)	7.9 (3.7–17.6)	81.2 (17.4–143.0)	61.3 (22.8–373.3)	45.6 (20.1–154.2)
		NOEC	15.6	125	312.5	125
		LOEC	31.25	250	625	250
	RL	EC_50_ (95% CI)	23.4 (13.2–31.6)	121.9 (98.4–151.3)	219.2 (123.3–289.6)	365.4 (222.9–462.8)
		EC_10_ (95% CI)	4.4 (2.6–17.5)	17.5 (10.0–65.5)	37.3 (18.6–114.4)	155.0 (50.9–174.8)
		NOEC	15.6	31.25	156.25	125
		LOEC	31.25	62.5	312.5	250
*L. minor*	FA	EC_50_ (95% CI)	166.2	>100	1756.1	>100
		EC_10_ (95% CI)	14.8 (5.4–33.6)	54.4 (23.1–195.7)	50.1 (32.7–145.9)	26.4 (17.8–80.2)
		NOEC	25	125	<125	<62.5
		LOEC	50	250	125	62.5
	RL	EC_50_ (95% CI)	40.8 (11.1–63.1)	155.1 (48.3–318.4)	109.2 (92.3–488.3)	145.3 (58.8–181.4)
		EC_10_ (95% CI)	3.4 (2.2–18.9)	13.5 (9.7–43.6)	21.8 (18.5–27.6)	15.7 (11.8–64.4)
		NOEC	25	125	<125	125
		LOEC	50	250	125	250

FA; frond area, RL; root length, EC_50_; the half-maximal effective concentration, NOEC; the highest concentration causing no-observable effect, LOEC; the lowest-observable-effect concentration.

**Table 2:** Summary statistics for SSDs fit to two duckweed test results (units: μg L^−1^).

	HC_05_	PNEC	NPSRW
Ag	0.0007	0.0001	NA
Cd	0.0678	0.0068	<5
Cr	0.1301	0.0130	<50
Cu	0.0441	0.0044	NA

HC_05_; the slope, PNEC; predicted no-effect concentration, NPSRW; Korean Nationally Permissible Standard for River Water. There are no criteria for Ag and Cu in the permissible limits of river water in Republic of Korea.

## Data Availability

All the results found are available in this manuscript.
